# Suture of the Flap, after Extract of Cataract

**Published:** 1867-04

**Authors:** Henry W. Williams


*Suture of the Flap, after extract of Cataract.* By Henry W. Williams, M. D.

(Read before the American Ophthalmological Society, June, 1866.)

I offer for the consideration of the Society a few suggestions respecting suture of the cornea after the removal of cataract by flap operation, in the hope that, by the adoption of such a modification of the ordinary procedure, we may so far lessen the risks of extraction that we shall not hereafter be tempted to incur the dangers attendant on the repeated introduction of instruments within the eye, and that mutilation of the iris may be rendered unnecessary.

My method consists in placing a single point of suture at the apex of the flap of the cornea, after extraction of the lens, and whilst the patient is still under the influence of ether.

After trial of various curved and straight needles, and of needles mounted upon a handle, I give the preference to straight needles of very minute size, less than a fourth of an inch in length, and with flat cutting points, as being best adapted to penetrate the corneal tissues. The objections to needles fixed upon a handle are, that it is difficult to disengage the extremely fine thread, and that on being withdrawn they drag upwards the corneal flap.

The great advantages claimed for this plan are as follows:

It renders etherization more applicable to extraction operations, in case emesis should occur; and the patient being thus impassive, the operator is enabled to do, with deliberation and care, whatever may be requisite in removing complications which may arise in course of an operation, without feeling he incurs a risk of contusing the iris or losing a portion of the vitreous during sudden involuntary movements of the eye. The edges of the wound being retained in close opposition, union by primary adhesion, the first desideratum in flap operations, is rendered much more certain. The puffy, swollen state of the margin of the flap, which renders the healing process difficult and uncertain, is thus avoided, and the eye resumes at once almost its normal condition. It nearly obviates all risk of spontaneous prolapse of the iris—the "*bete noire*," to use the words of Mr. Dixon, "of extraction of operations." By effecting a speedy re-establishment of the anterior chamber, it admits of the free use of atropia, without fear that *prolapsus iridis* may ensue, thus allowing continued dilatation of the pupil to be kept up, and lessening the risk of irritation of the iris from unremoved fragments of the lens, or torn edges of capsule, or from proliferous degeneration of the intracapsular cells. It permits of early and frequent inspection of the eye, and the prompt discovery of any morbid phenomena, so that timely recourse may be had to appropriate remedies.

It much abbreviates the term of rigorous confinement of the patient, and shortens the entire period of convalescence.

A single strand of the finest silk is employed for the suture. The needle is seized with strong forceps, and passed through the edges of the wound, which are held with very delicate toothed forceps. The eye being entirely passive, the manœuvres may be executed with delicacy and without haste. Gentle compression, by means of lint and a flannel bandage, constitutes the after-treatment.

In most cases the suture has been left to come away of itself, and, though usually becoming detached within a few days, it has in some instances remained *in situ* for seven weeks, without giving rise to more than trivial irritation. I am satisfied, however, that its presence for a longer period than is necessary is undesirable, and serves slightly to retard the parient's recovery. My present practice is to administer ether, and remove the suture within a week after the operation, if it has not sooner been eliminated. It is unsafe to attempt its removal except during anæsthesia, as a sudden move of the globe, or the pressure of the forceps, if fixation be resorted to, might, as in one of my own cases, cause a re-opening of the wound.

In no instance, so far as I could judge, has the suture given rise to any serious symptoms. In twenty-four cases subjected to this treatment, there have only been two failures.—*Detroit Rev. Med.*

